# Pulmonary Flow Management by Combination Therapy of Hemostatic Clipping and Balloon Angioplasty for Right Ventricular-Pulmonary Artery Shunt in Hypoplastic Left Heart Syndrome

**DOI:** 10.1007/s00246-024-03579-6

**Published:** 2024-07-19

**Authors:** Yusuke Shigemitsu, Maiko Kondo, Yoshihiko Kurita, Yosuke Fukushima, Yuya Kawamoto, Kenta Hirai, Mayuko Hara, Tomoyuki Kanazawa, Tatsuo Iwasaki, Shingo Kasahara, Koichi Kataoka, Hirokazu Tsukahara, Kenji Baba

**Affiliations:** 1 Department of Pediatrics, Okayama University Hospital, 2-5-1, Shikata-Cho, Kita-Ku, Okayama-Shi, Okayama, 700-8558 Japan; 2 Department of Pediatric Anesthesiology, Okayama University Hospital, Okayama, Japan; 3 Department of Cardiovascular Surgery, Okayama University Hospital, Okayama, Japan; 4 Department of Pediatric Cardiology, Hirohima City Hiroshima Citizens Hospital, Hiroshima, Japan

**Keywords:** Hypoplastic left heart syndrome, Norwood palliation, Balloon angioplasty, Congenital heart disease

## Abstract

Controlling pulmonary blood flow in patients who have undergone Norwood palliation, especially early postoperatively, is challenging due to a change in the balance of systemic and pulmonary vascular resistance. We applied a combination therapy of clipping and balloon angioplasty for right ventricle—pulmonary artery (RV-PA) shunt to control pulmonary blood flow, but the influence of the combination therapy on the PA condition is uncertain. Retrospectively analysis was conducted of all infants with hypoplastic left heart syndrome who had undergone Norwood palliation with RV-PA shunt at Okayama University Hospital from January 2008 to September 2022. A total of 50 consecutive patients underwent Norwood palliation with RV-PA shunt in this study period. Of them, 29 patients underwent RV-PA shunt flow clipping, and the remaining 21 had unclipped RV-PA shunt. Twenty-three patients underwent balloon angioplasty for RV-PA shunt with clips. After balloon angioplasty, oxygen saturation significantly increased from 69 (59–76)% to 80 (72–86)% (*p* < 0.001), and the narrowest portion of the clipped conduit significantly improved from 2.8 (1.8–3.4) to 3.8 (2.9–4.6) mm (*p* < 0.001). In cardiac catheterizations prior to Bidirectional cavo-pulmonary shunt (BCPS), there were no significant differences in pulmonary-to-systemic flow ratio (Qp/Qs), ventricular end-diastolic pressure, Nakata index, arterial saturation, mean pulmonary artery pressure and pulmonary vascular resistance index. On the other hand, in Cardiac catheterizations prior to Fontan, Nakata index was larger in the clipped group (*p* = 0.02). There was no statistically significant difference in the 5-year survival between the two groups (clipped group 96%, unclipped group 74%, log-rank test: *p* = 0.13). At least, our combination therapy of clipping and balloon angioplasty for RV-PA shunt did not negatively impact PA growth. Although there is a trend toward better but not statistically significant difference in outcomes in the clipped group compared to the non-clipped group, this treatment strategy may play an important role in improving outcomes in hypoplastic left heart syndrome.

## Introduction

The surgical outcome of patients with hypoplastic left heart syndrome (HLHS) has dramatically improved in recent years [1, 2]. The principle of Norwood palliation of patients with HLHS is to secure unobstructed systemic outflow, unobstructed intra-atrial communication, and controlled pulmonary blood flow. It is challenging to control pulmonary blood flow, especially early postoperatively, due to systemic and pulmonary vascular resistance balance changes. Excessive pulmonary blood flow results in systemic hypoperfusion, and inadequate pulmonary circulation causes severe hypoxemia. Adjustable systemic-pulmonary shunt technique using hemostatic clip or tourniquet to prevent pulmonary overcirculation in early postoperative stage were reported [3–6]. We applied clipping and balloon angioplasty (BAP) therapy for the right ventricle—pulmonary artery (RV-PA) shunt to control pulmonary blood flow. Our concept is that restrictive pulmonary flow by clipping contributes to the stability of hemodynamics at the early period and that BAP for clipped RV-PA shunt improves infants’ oxygen saturation and pulmonary vascular bed. In this study, we reviewed our experiences to evaluate the combination therapy of clipping and BAP for RV-PA shunt, comparing the infants with unclipped RV-PA shunt.

## Methods

Retrospectively analysis was conducted of all infants with HLHS who had undergone Norwood palliation with RV-PA shunt at Okayama University Hospital from January 2008 to September 2022. The Research Ethics Board at Okayama University Hospital approved this study (#2403–013), and informed consent was obtained via opt-out due to the retrospective nature of this study.

### Surgical Techniques

The technique of Norwood palliation with RV-PA shunt has been described before [7, 8]. A valveless RV-PA conduit was constructed using expanded polytetrafluoroethylene (e-PTFE). A 5 mm RV-PA conduit was used for patients weighing 3.5 kg or less, and 6 mm for those more than 3.5 kg. Delayed sternal closure was performed in all patients after Norwood procedure.

### Indication for Clipping of RV-PA Shunt

**Fig. 1:**
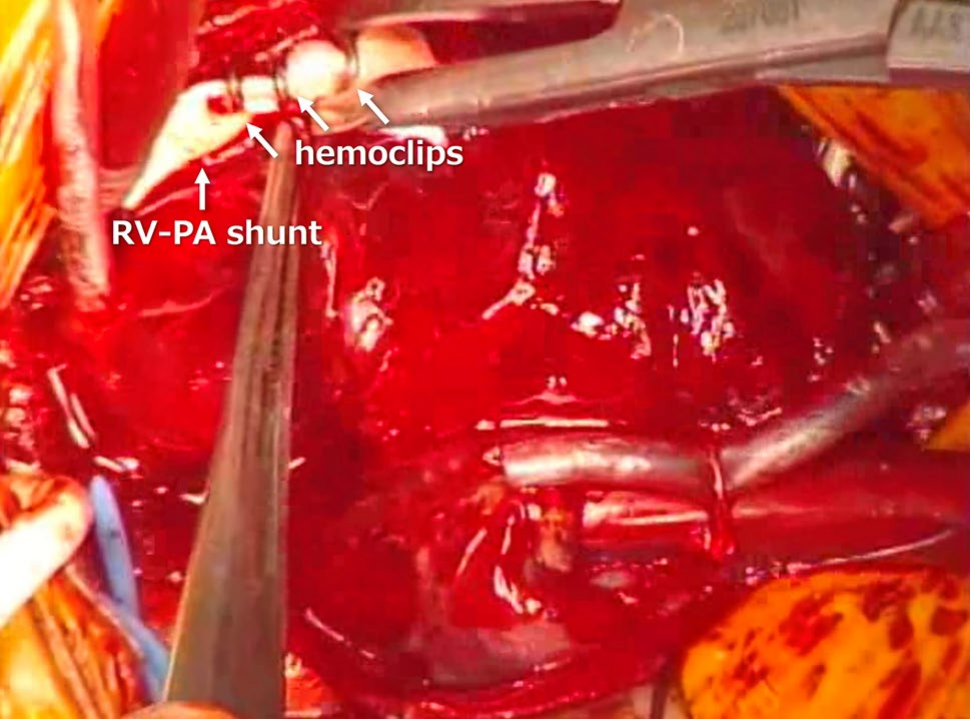
Intraoperative picture. In this case, 3 hemoclips were placed on the RV-PA shunt

When systemic oxygen saturation remained higher than 85% with inspired oxygen concentration of 21 to 40% despite ventilatory support or medical therapy to prevent pulmonary overcirculation, M-sized hemoclips for hemostasis were placed on the RV-PA shunt at the time of cessation of cardiopulmonary bypass for Norwood palliation or delayed sternal closure. RV-PA shunt was lightly clipped to facilitate easy detachment during subsequent BAP (Fig. 1). The target arterial oxygen saturations of clipping RV-PA shunt were 75–85% on fractional inspired oxygen of 21–40%.

### Cardiac Catheterization Technique and Angiographic Assessment

**Fig. 2:**
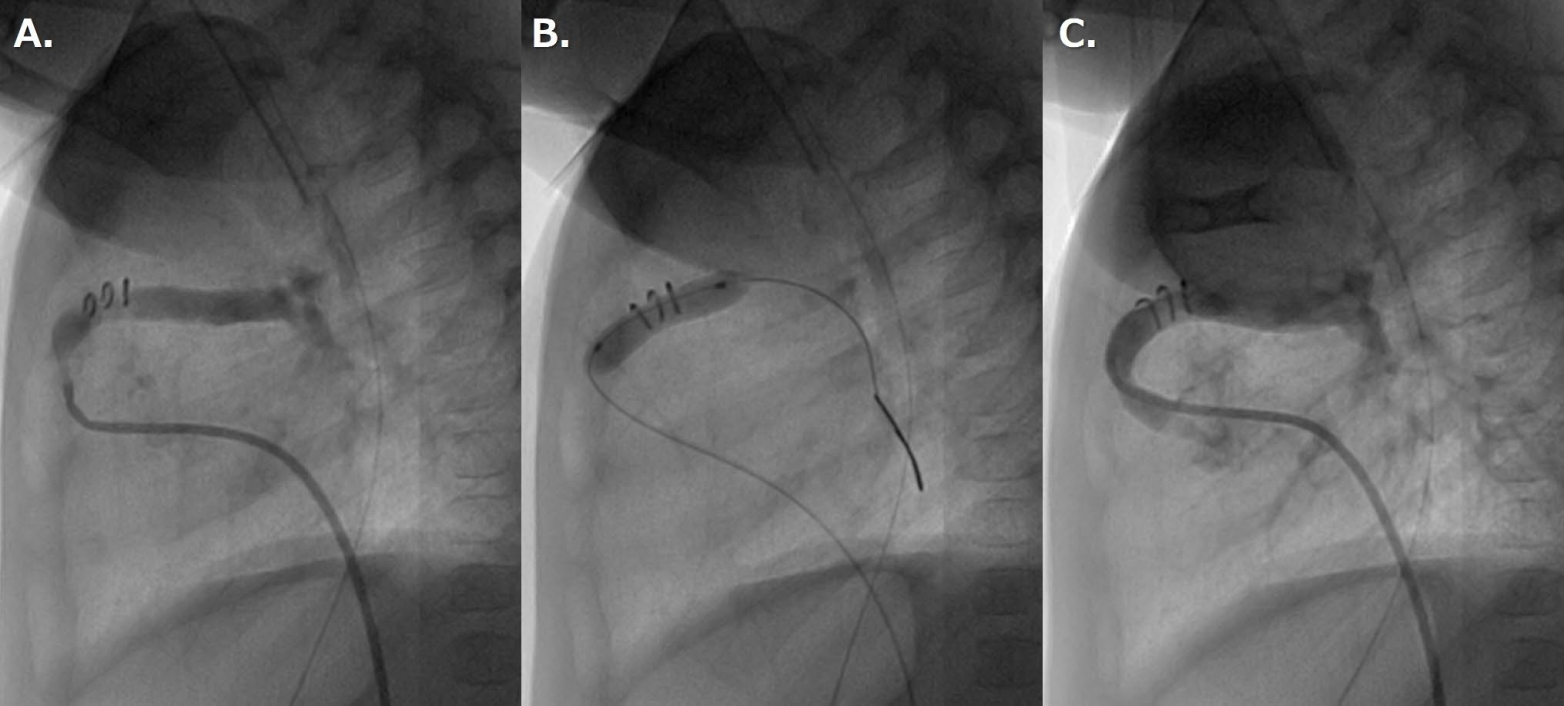
Lateral RV-PA shunt angiography in the same case as Fig. 1. **A** pre dilatation, **B** during dilatation, and **C** post dilatation

All procedures were performed under general anesthesia. The RV-PA shunt and pulmonary artery were approached from the femoral vein in all cases. A 4-French Balloon Angiographic Catheter with an end-hole (Gadelius Medical, Tokyo, Japan) was inflated to pass through the tricuspid valve and advanced through the RV-PA shunt. If the approach with the balloon catheter was challenging, the wire was used to exchange it for a 4-French Judkins right coronary catheter after passing through the tricuspid valve. The hemodynamic studies and biplane angiograms in antero-posterior and lateral projection were obtained. Based on the angiography assessment, a balloon catheter was chosen. A 0.014″–0.018″ inch hydrophilic guide wire was introduced into the left or right pulmonary artery via the catheter, the balloon catheter was advanced into the RV-PA shunt over the guide wire, and the BAP for the RV-PA shunt was performed (Fig. 2). After BAP, the hemodynamic data and angiograms were collected again. A Nakata index was calculated as the sum of the right and left PA cross-sectional areas indexed to the patient’s body surface area.

### Statistical Analysis

Data are expressed as median with range. Continuous variables were compared using the Mann Whitney test. Dichotomous and categoric variables were analyzed using Fisher’s exact test and chi-square test. Probability values less than 0.05 were considered statistically significant.

## Results

### Patient Characteristics

A total of 50 consecutive patients underwent Norwood palliation with RV-PA shunt in this study period. Of them, 29 patients underwent RV-PA shunt flow clipping, and the remaining 21 had unclipped RV-PA shunt. Of the 29 patients in the clipped group and 21 in the unclipped group, 8 (28%) and 9 (43%) underwent bilateral pulmonary artery banding (PAB) prior to Norwood palliation with RV-PA shunt due to high-risk factors, including low birth weight, prematurity, preoperative cerebral hemorrhage, organ failure and more than moderate tricuspid valve regurgitation. The case with chromosome abnormalities was observed in only one individual (3%) from the clipped group. In the clipped group, there were no complications related to clipping the shunt, such as acutely severe cyanosis. Of the 29 patients in the clipped group, 23 (79%) patients underwent BAP for RV-PA shunt with clips, and the remaining 6 (21%) patients did not undergo BAP for the clips. The patient characteristics and diagnosis are listed in Table 1.

**Table 1:** Patient characteristics and diagnosis

Variables	Clipped group	Unclipped group	*p* value
	(*n *= 29)	(*n *= 21)	
Male gender (%)	17 (59)	12 (57)	0.73
Birth weight (kg)	3.0 (2.1–3.6)	2.8 (2.2–3.6)	0.50
Chromosome abnormality (%)	1 (3)	0 (0)	NS
Bilateral PAB (%)	8 (28)	9 (43)	0.12
Age at Norwood (d)	4 (1–64)	25 (3–129)	0.01
Weight at Norwood (kg)	3.1 (2.3–3.8)	3.2 (2.6–4.1)	0.07
Hypoplastic left heart syndrome			
MA/AA	18	14	
MA/AS	3	0	
MS/AA	6	5	
MS/AS	2	2	

### Intensive Care Unit Stays After Norwood Palliation

The intensive care unit stays after Norwood procedure were 16 (10–33) days in the clipped group and 15 (7–37) days in the unclipped group, respectively. No significant difference was found between the two groups (*p* = 0.91).

### BAP for Clipped RV-PA Shunt

Twenty-three patients underwent BAP for RV-PA shunt with clips at a median age of 141 (20–305) days and a median weight of 5.1 (2.9–6.7) kg; 20 patients underwent one procedure, and three patients underwent two procedures. The balloon catheter we used was Sterling (Boston Scientific, MA, USA), Lacrosse (Goodman, Nagoya, Japan), or Sapphire II pro PTA (OrbusNeich, Hong Kong, China) of 3.5–6 mm in diameter, which are shown in Table 2. The diameter of the selected balloon was generally equal to or 1 to 1.5 mm smaller than the RV-PA shunt size.

**Table 2:** Balloon size

Diameter(mm)	Patients
3.5	1
4	6
5	14
6	2
Same diameter as RV-PA shunt	16
Shunt—1mm	6
Shunt—1.5mm	1

**Fig. 3:**
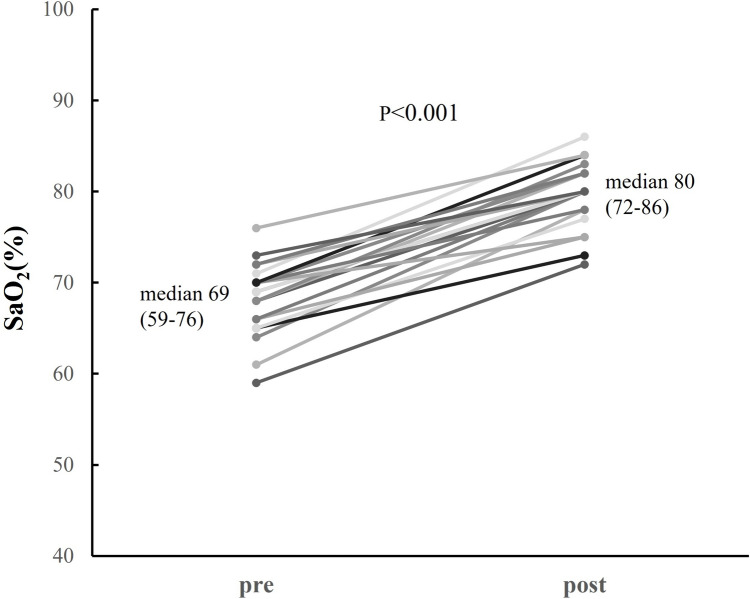
The changes of SaO2 before and after BAP. SaO2 significantly increased from 69 (59–76) % to 80 (72–86) %

**Fig. 4:**
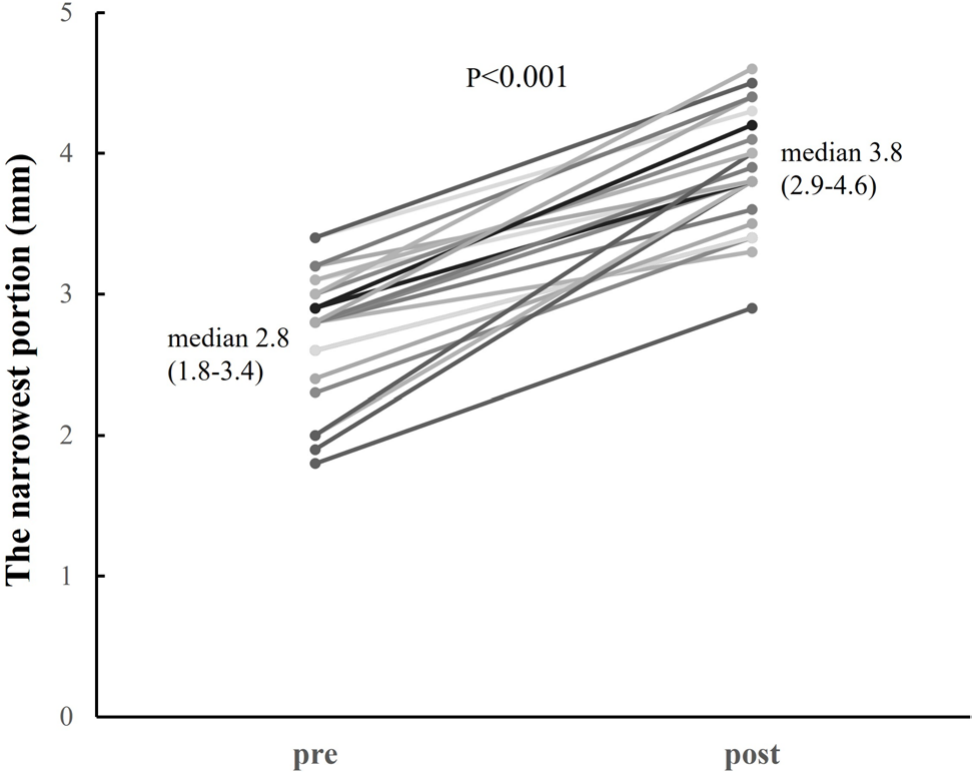
The changes of the narrowest portion diameter before and after BAP. The diameter significantly improved from 2.8 (1.8–3.4) mm to 3.8 (2.9–4.6) mm

After BAP, oxygen saturation significantly increased from 69 (59–76) % to 80 (72–86) % (*p* < 0.001), and the narrowest portion of the clipped conduit significantly improved from 2.8 (1.8–3.4) mm to 3.8 (2.9–4.6) mm (*p* < 0.001). (Figs. 3 and 4).

A complication of BAP for clipped RV-PA shunt occurred in 5 of 23 patients (22%). Of them, one patient had a transient complete atrioventricular block during the procedure. The remaining four patients worsened the tricuspid regurgitation due to the rapid increase in pulmonary blood flow and subsequent right ventricular volume overload, all of which were clinically acceptable. Any other complications attributable to pulmonary overcirculation, such as renal dysfunction, necrotizing enterocolitis, needing more inotropic support, were not observed.

The concomitant interventions with BAP for clipped RV-PA shunt included 12 BAPs for PA (52%) and 5 BAPs for aortic arch (22%). It should be noted that stent implantation for RV-PA shunt has not been performed in our center.

### Pre BCPS Evaluation

Cardiac catheterizations prior to BCPS were performed at a mean age of 153 (90–305) days in the clipped cohort and 143 (86–258) days in the unclipped cohort, with no significant difference observed between the two groups (*p* = 0.75). Among the 23 patients in the clipped group who underwent BAP, 20 had a pre-BCPS evaluation at the same session as the BAP, and 3 had a pre-BCPS evaluation at a separate session after the BAP. Pulmonary-to-systemic flow ratio (Qp/Qs), ventricular end-diastolic pressure, Nakata index, arterial saturation, mean pulmonary artery pressure, and pulmonary vascular resistance index were comparable between the clipped and unclipped cohort. There were no significant differences in any variables (Table 3).

**Table 3:** Pre-BCPS evaluation

Variables	Clipped group	Unclipped group	*p* value
	(*n *= 28)	(*n *= 18)	
Body weight (kg)	5.1 (4.0–6.7)	5.0 (2.6–5.8)	0.23
Body surface area (m^2^)	0.28 (0.24–0.35)	0.29 (0.19–0.32)	0.56
Qp/Qs	0.69 (0.34–1.87)	0.73 (0.33–1.30)	0.48
RVEDP (mmHg)	6 (4–14)	6 (4–8)	0.54
Nakata index (mm^2^/m^2^)	201 (126–334)	187 (101–304)	0.31
Arterial saturation (%)	72 (63–88)	72 (64–80)	0.23
Mean pulmonary artery pressure (mmHg)	12 (9–21)	13 (9–20)	0.81
Pulmonary vascular resistance index (Woods Units m^2^)	1.53 (0.51–3.87)	1.85 (0.60–2.80)	0.57

### Pre Fontan Evaluation

Cardiac catheterizations prior to Fontan were performed at a mean age of 671 (517–1256) days in the clipped cohort and 768 (488–2247) days in the unclipped cohort, with no significant difference observed between the two groups (*p* = 0.11). Qp/Qs, ventricular end-diastolic pressure, arterial saturation, mean pulmonary artery pressure, and pulmonary vascular resistance index were comparable between the groups. Nakata index was larger in the clipped group (*p* = 0.02) (Table 4).

**Table 4:** Pre-Fontan evaluation

Variables	Clipped group	Unclipped group	*p* value
	(*n *= 24)	(*n *= 15)	
Body weight (kg)	9.8 (8.2–13.0)	10.1 (7.9–15.0)	0.92
Body surface area (m^2^)	0.46 (0.41–0.59)	0.45 (0.42–0.68)	0.92
Qp/Qs	0.62 (0.44–0.78)	0.58 (0.45–0.87)	0.32
RVEDP (mmHg)	6 (5–8)	6 (4–9)	NS
Nakata index (mm^2^/m^2^)	228 (166–395)	194 (143–270)	0.02
Arterial saturation (%)	83 (69–89)	82 (75–90)	0.42
Mean pulmonary artery pressure (mmHg)	11 (8–15)	11 (7–14)	0.96
Pulmonary vascular resistance index (Woods Units m^2^)	1.55 (0.50–2.59)	1.84 (0.80–2.80)	0.26

### Outcomes

**Fig. 5:**
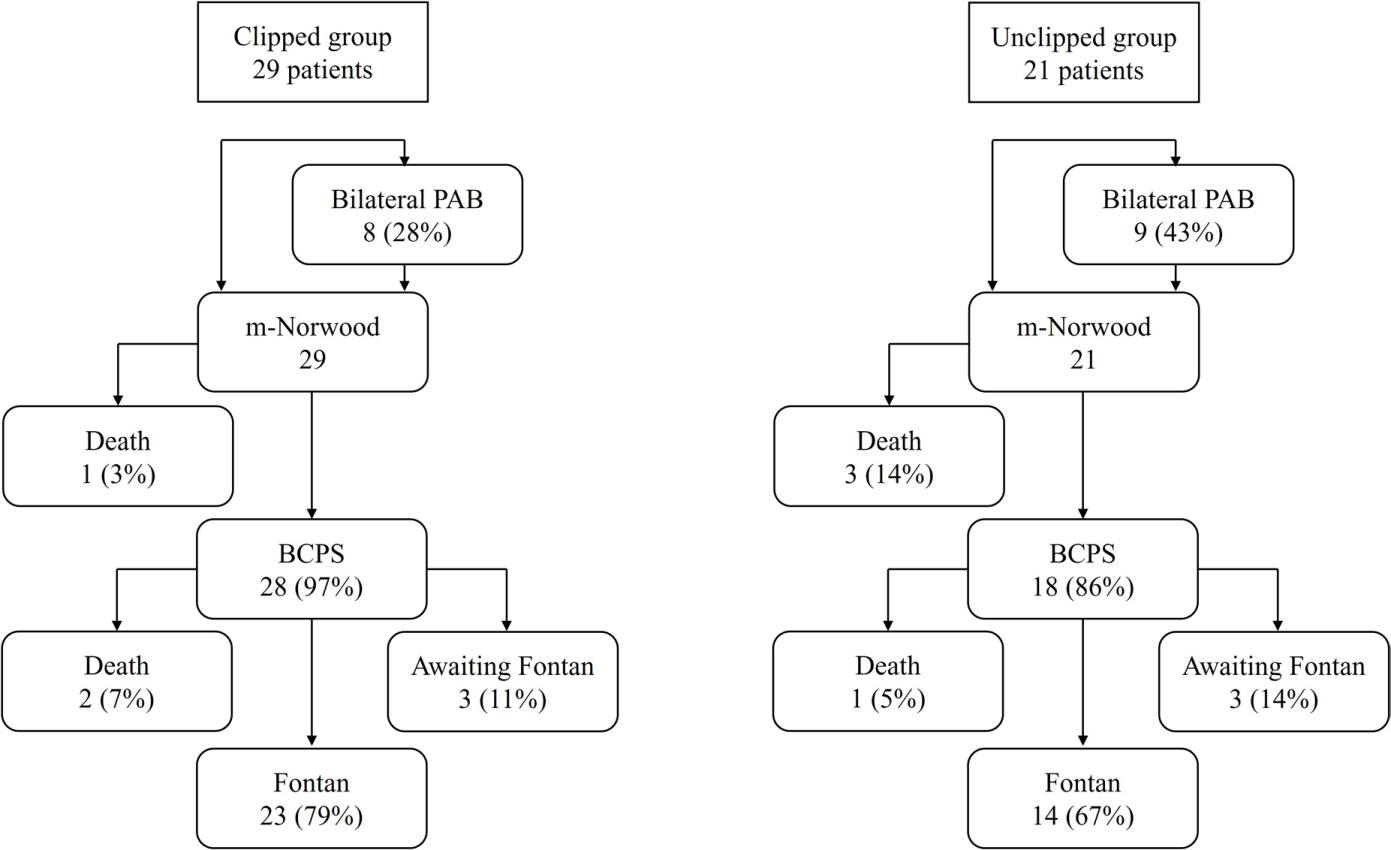
Overall outcomes for the clipped and unclipped groups

**Fig. 6:**
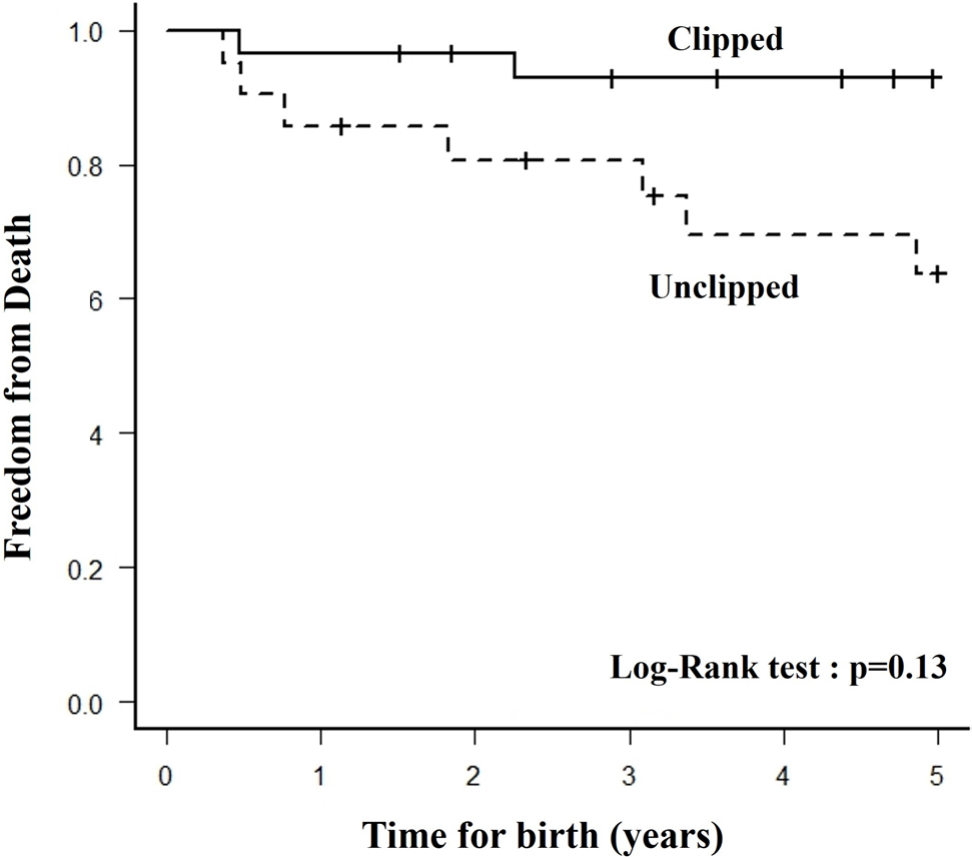
Kaplan-Maier analysis for freedom from death between the clipped group and the unclipped group. There is no statistically significant deference (Log-rank test: *p* = 0.13)

Of the 29 patients in the clipped group and 21 patients in the unclipped group, 28 (97%) and 18 (86%) underwent BCPS. No statistically significant difference was found in survival to BCPS between the groups (*p* = 0.30). At the last follow-up, three patients in the clipped group and three patients in the unclipped group were awaiting Fontan procedure, and 23 patients in the clipped group and 14 in the unclipped group had undergone Fontan procedure (Fig. 5). The Kaplan–Meier curve for freedom from death is shown in Fig. 6, with no statistically significant difference in the 5-year survival between the two groups (clipped group 96%, unclipped group 74%, log-rank test: *p* = 0.13).

## Discussion

In parallel circulation, such as Norwood palliation of patients with HLHS, it is vital to maintain a balance between systemic and pulmonary circulation. The treatment of circulatory collapse just after Norwood palliation due to excessive pulmonary blood flow has included respiratory management with CO_2_ accumulation, has been used to increase pulmonary vascular resistance [9], and vasodilators like sodium nitroprusside have been used to decrease systemic vascular resistance. As it takes time for respiratory management and drug administration to be effective, there have been previous reports [3, 4] of acute postoperative circulatory improvement using a tourniquet or clip restriction of a modified BT shunt in cases of excessive pulmonary blood flow immediately after the Norwood palliation.

In our center, the RV-PA shunt has been used for Norwood surgery for HLHS since 1996, achieving good results [7]. The RV-PA shunt with Norwood has been reported to be more effective than the modified BT shunt with Norwood in securing systemic circulation in the acute postoperative period [8]. To further improve the outcome, since 2008, we have adopted a combination therapy in which the RV-PA shunt is clipped, followed by unclipping by catheterization if needed. In this article, we review the clinical results of the combination therapy.

The goal of the good Fontan candidate is to maintain cardiac and atrioventricular valve function and an adequate pulmonary vascular bed. Extensive Qp/Qs could lead to cardiac dysfunction and progressive atrioventricular valve regurgitation. On the other hand, a low Qp/Qs leads to progressive cyanosis, which results in insufficient pulmonary vascular bed. A good Fontan candidate must maintain an appropriate Qp/Qs balance. Due to the artificial structure, the RV-PA shunt is relatively undersized with somatic growth, so it would be reasonable to increase pulmonary blood flow by unclipping before BCPS palliation to maintain optimal Qp/Qs.

It was reported that RV-PA shunts are restricted by tourniquet [3] or clip [4, 5] at the time of Norwood palliation, and the restriction is removed at the time of delayed sternal closure, which is performed 2–5 days after the Norwood procedure. Yasukawa [6] et al. reported that a clip is placed on the RV-PA shunt to restrict pulmonary blood flow while the sternum is open before delayed sternal closure, followed by unclipping by catheter balloon dilation, if necessary. Likewise, at our institution, if pulmonary overcirculation is suspected at the cessation of cardiopulmonary bypass for Norwood palliation or delayed sternal closure, the RV-PA shunt is clipped to restrict pulmonary blood flow.

Intensive care unit stays were equivalent in the clipping and non-clipping groups, although clipping was placed on RV-PA shunts in patients suffering from pulmonary overcirculation. This suggests that clipping might have contributed to hemodynamic stability in the acute postoperative period.

Subsequently, when the cyanosis worsens, catheter balloon dilation is performed for unclipping. The balloon size was reported [6] as body weight (kg) plus 1 mm for partial unclipping and RV-PA conduit size for full unclipping.

High-risk HLHS patients often undergo bilateral PAB prior to the Norwood palliation [10], and patients who underwent bilateral PAB have been reported to require more surgical or catheter interventions on the pulmonary arteries due to pulmonary artery stenosis [11]. There were 8 of 29 patients in the clip group and 9 of 21 in the non-clip group who underwent bilateral PAB prior to Norwood palliation. Although this difference was not statistically significant, the large number of non-clipped groups might explain the significantly later timing of Norwood palliation and the significantly smaller Nakata index at the pre-Fontan evaluation compared to the clipped group.

In our treatment strategy, in the case of bilateral PAB for high-risk HLHS patients, the next palliation is supposed to be the Norwood procedure, as the so-called comprehensive stage II (Norwood + BCPS) is extremely rare. Therefore, prostaglandin E1 is administered for the ductus arteriosus instead of PDA stenting. It should be noted that comprehensive stage II was performed in only three patients excluded from the present study due to the absence of RV-PA shunt placement.

There have been reports [12–14] of transient arrhythmias and transient complete atrioventricular block occurring during BAP and stent placement for RV-PA shunt with Norwood. In our series, a transient complete atrioventricular block during unclipping was observed in one patient, but he recovered quickly and did not require treatment. Mah et al. described [14] that a catheter from the inferior vena cava a loop to push against the ventricular septum, mechanically irritating the conduction system, and that physicians should have an increased awareness of the potential for catheter-induced heart block in these patients.

As for complications associated with unclipping for the RV-PA shunt, in our case, worsening of tricuspid regurgitation was observed in 4 of 23 patients who underwent unclipping, but it was clinically acceptable. Unclipping increases pulmonary blood flow and, thus, right ventricular volume overload, which could lead to worsening tricuspid regurgitation. There is, however, a report [6] of urgent BCPS due to right heart failure caused by increased tricuspid regurgitation immediately after unclipping for the RV-PA shunt. The tricuspid valve may have been mechanically injured during the balloon dilatation for unclipping. Therefore, catheter interventionalists should ensure the end-hole balloon catheter is inflated to avoid entering RV through an inter-chordal space. As for our cases with worsening tricuspid regurgitation after unclipping, no cases of mechanical tricuspid valve injury were found, and all were thought to be due to increased right ventricular volume overload.

## Study Limitations

This study is a retrospective, non-randomized review of a small cohort from a single institution, and thus suffers from limitations intrinsic to those boundaries. The different percentage of patients who underwent bilateral PAB between the two groups might have affected the comparisons. The follow-up period of this cohort is relatively short, so further follow-up studies are necessary.

## Conclusions

A combination therapy of clipping and BAP for RV-PA shunt was performed for the HLHS patients suffering from pulmonary overcirculation. The ICU stay after Norwood palliation in the clipped group was comparable to that in the non-clipped group, indicating the effect of clipping in the acute post operative period. As the clipped group showed no inferiority in PA growth and mortality compared to the unclipped group, our combination therapy is considered useful as one of the management methods in HLHS.

## Data Availability

No datasets were generated or analysed during the current study.
